# Not a Common Rectosigmoid Perforation

**DOI:** 10.7759/cureus.1465

**Published:** 2017-07-13

**Authors:** Rizwan Ishtiaq, Khurram Niaz, Darakshan Masood, Daniyal Ishtiaq, Shafay Yasin, Umer H Chaudhry, Anam Ashraf

**Affiliations:** 1 Gastroenterology, Beth Israel Deaconess Medical Center, Boston; 2 Department of General Surgery, Bahawal Victoria Hospital, Bahawalpur, Pakistan; 3 Department of Gynecology, Bahawal Victoria Hospital, Bahawalpur, Pakistan; 4 General Medicine, Rawalpindi Medical College, Rawalpindi, Pakistan; 5 Internal Medicine, Rawalpindi Medical College, Rawalpindi, Pakistan

**Keywords:** geophagia, rectosigmoid, perforation, mud

## Abstract

Gastrointestinal perforation is a common complication arising due to homicidal injuries, trauma or intake of medications like aspirin. Intestinal perforation caused by chronic intake of mud, clay or soil is a rare phenomenon and very few cases have been reported in the literature. We hereby present the first case of rectosigmoid perforation from Pakistan which was caused by chronic mud intake in a female patient. Diagnosis of this condition in its early stage is important because it can be fatal if not addressed urgently.

## Introduction

Geophagia is defined as an intentional consumption of mud, clay or soil [1]. It has been coined as a psychiatric illness from various points of view. Repair of gastrointestinal perforation is a common practice in surgery. Use of medications such as aspirin or non-steroidal anti-inflammatory drugs, stab injuries, gunshots and blunt trauma to the abdomen are common causes of gastrointestinal perforation [2]. Typical symptoms include fever, severe abdominal pain, constipation, nausea and vomiting. Constipation is usually a benign symptom in the presentation of gastrointestinal perforation. We present the first case of rectosigmoid perforation from Pakistan in a 50-year-old female patient which was caused by chronic intake of mud. Informed consent was obtained from the patient.

## Case presentation

A 50-year-old female presented with signs and symptoms of constipation, nausea, vomiting, pain in abdomen with swelling and unable to pass flatus for the last 12 hours. Based on her X-ray findings of colonic distention and collapsed distal colon, a diagnosis of large bowel obstruction was made. She had a history of reporting to the emergency room with signs and symptoms of subacute obstruction. According to the patient’s son, the patient had a habit of eating mud for a long time. The doctors did not notice this fact in her previous visits to the emergency room. The patient had no history of psychiatric illness, hypertension or diabetes.

**Figure 1:**
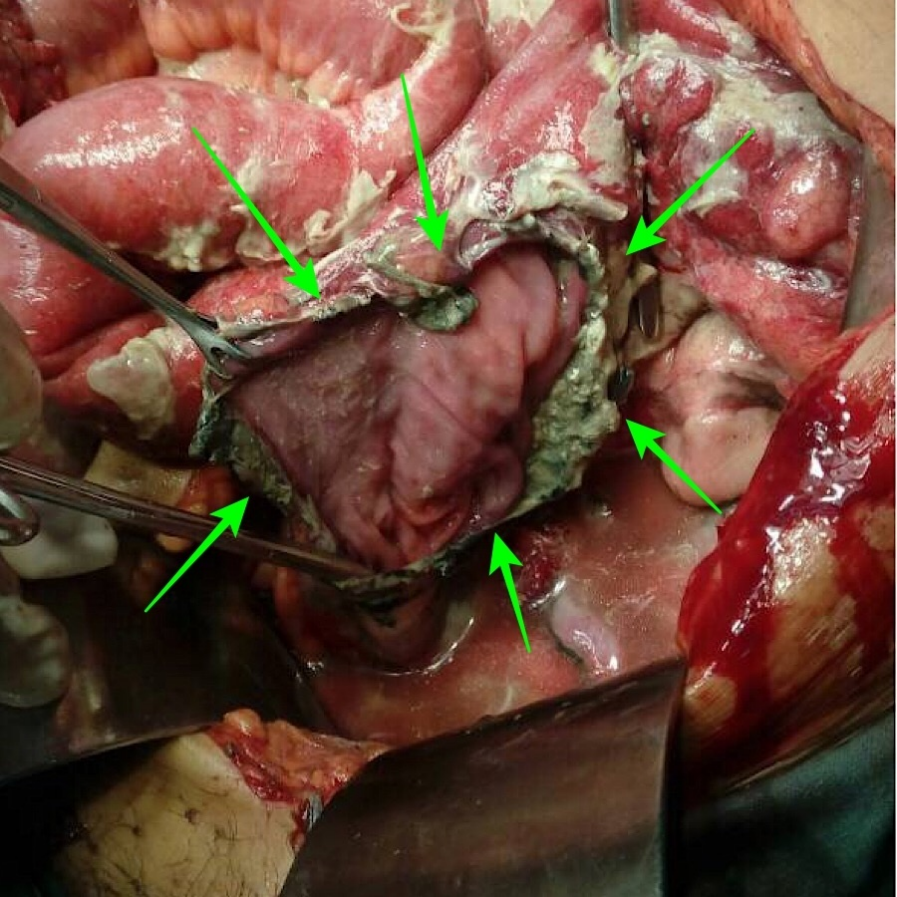
Rectosigmoid perforation evident by the green arrows.

**Figure 2:**
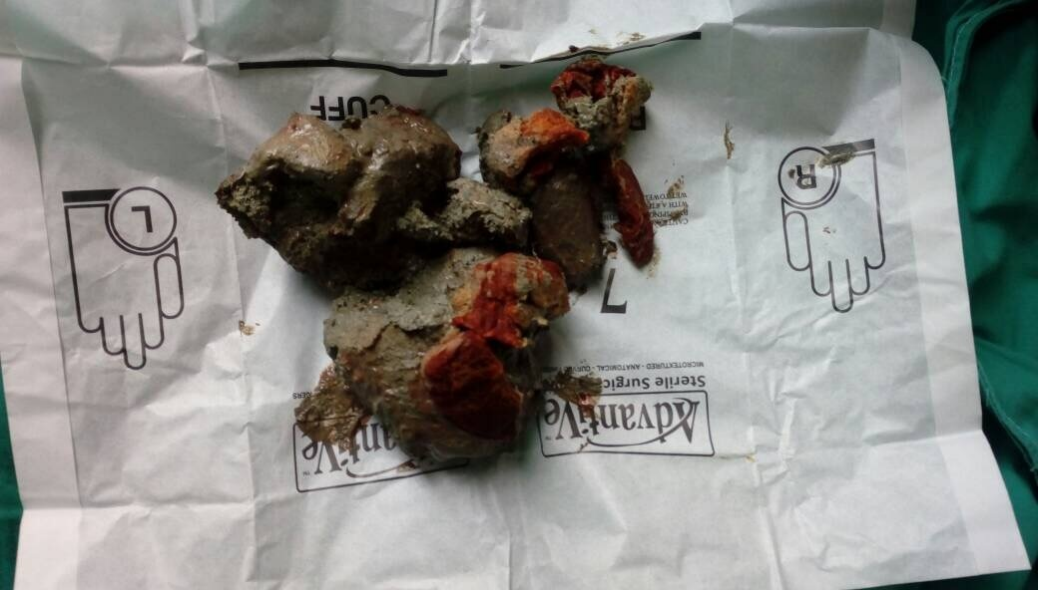
A collection of mud obtained from the patient during exploratory laparotomy.

The patient was transferred to the operating room and exploratory laparotomy was performed to relieve the obstruction. During the procedure, perforation was found on the anterior wall at the rectosigmoid junction and mud residing outside in the peritoneal cavity. Figure 1 and Figure 2 refer to the perforation of the rectosigmoid junction and the collection of mud during the surgery respectively.

 

Hartmann’s procedure was performed. Postoperatively, she went into prolonged ileus. Laboratory investigations did not reveal iron deficiency anemia and were completely normal. Re-exploration was performed and stoma revised after removing mud from transverse colon. The patient ultimately recovered after one month. She is scheduled for the reversal of Hartmann’s procedure after three months.

## Discussion

Perforation of rectum or sigmoid is not an uncommon entity. It can occur in any age group. Rectosigmoid colon is characterized by inadequate blood supply and high pressure due to less caliber which is the rationale behind perforation of the distal part of the colon [3]. Our case is the first case of rectosigmoid perforation caused by geophagia from Pakistan reported in the literature. Previously, a few cases of geophagia causing rectosigmoid perforation have been reported in the literature. A case of sigmoid colon perforation was reported in a nonpregnant African woman due to geophagia [4]. Hawass, et al. presented the report of three cases with geophagia [1]. Two cases had a history of mud intake whereas the third case had a history of eating pebbles. Macheka, et al. studied the prevalence and factors leading to geophagia in pregnant women in Pretoria, South Africa [5]. In their study of 597 pregnant women, 54% (n = 323) were documented with geophagia. Almost half of these, 323 pregnant women had an unexplained craving for the soil consumption. Eighty-four patients had to crave due to the smell of soil before the rain. Poverty was linked with geophagia as almost 237 women with geophagia were unemployed. Literacy was not found to play a key role as both literate and illiterate women were found as consumers of soil.

Chronic intake of mud or clay can be attributed to pica which is associated with iron deficiency anemia and obsessive compulsive disorder [6-7]. Our patient had no laboratory evidence of iron deficiency anemia or past medical history of obsessive compulsive disorder which also makes our case unique. Our patient presented to the emergency in the past with signs of subacute obstruction but due to lack of proper history and exposure to such cases, consideration of geophagia causing gastrointestinal obstruction or perforation was ignored.

Surgical exploration is the only definitive treatment in such cases. Mortality rate is higher in cases of infection, septic shock or delayed treatment [8].

## Conclusions

Rectosigmoid perforation is a frequent source of acute abdomen and should always be kept in the differentials when a patient presents with severe abdominal pain and constipation. Patients presenting with similar signs and symptoms in frequent visits should be investigated properly. Geophagia can also lead to gastrointestinal obstruction or perforation. It is important for physicians to be aware of this condition especially in patients who are malnourished or belong to a very poor economic class.
